# Sildenafil for Attenuating Hepatic Ischemia-Reperfusion Injury and Promoting Liver Regeneration After Major (65%) Hepatectomy: A Porcine Pilot Study

**DOI:** 10.7759/cureus.110708

**Published:** 2026-06-11

**Authors:** Georgios Gemenetzis, Apostolos Papalois, Vassilios Smyrniotis, Nikolaos Arkadopoulos, Panteleimon Vassiliu

**Affiliations:** 1 Department of Surgery, National and Kapodistrian University of Athens, Athens, GRC; 2 Department of Translational Research and Training, Experimental, Educational and Research Centre ELPEN Pharmaceutical Co. Inc., Athens, GRC; 3 Fourth Department of Surgery, Attikon University Hospital, National and Kapodistrian University of Athens, Athens, GRC; 4 Department of Surgery, Fourth Surgical Clinic, Attikon University Hospital, National and Kapodistrian University of Athens, Athens, GRC

**Keywords:** hepatectomy, hepatic ischemia-reperfusion injury, liver regeneration, phosphodiesterase-5 inhibitor, porcine model, post-hepatectomy liver failure, sildenafil

## Abstract

Introduction

Hepatic ischemia-reperfusion (I/R) injury is a major driver of post-hepatectomy liver failure (PHLF) and contributes to morbidity after major liver resection. Sildenafil, a selective phosphodiesterase-5 (PDE-5) inhibitor, has shown protective effects against I/R injury in cardiac, renal, and rodent hepatic models, but pre-clinical data in large animals undergoing major hepatectomy are scarce. The aim of this study is to evaluate whether perioperative oral sildenafil attenuates I/R injury and enhances liver regeneration after 65% hepatectomy combined with 45 minutes of warm ischemia in a porcine model.

Methods

Fourteen male domestic pigs (28-39 kg) were randomly allocated to two groups of seven. Animals in the control group (Group A) underwent 65% hepatectomy with 45 minutes of continuous portal triad clamping (Pringle maneuver). Animals in the sildenafil group (Group B) underwent the same operation but additionally received oral sildenafil 0.3 mg/kg every eight hours, beginning 24 hours before surgery and continuing for seven postoperative days. Hemodynamic parameters, arterial blood gases, liver function tests (including bilirubin and markers of hepatocellular injury (ALT, AST, LDH), prothrombin time (PT), aPTT), and inflammatory markers (tumor necrosis factor-α (TNF-α), IL-1, IL-6, malondialdehyde (MDA), C-reactive protein) were measured at predefined time points up to 24 hours from reperfusion and on postoperative day 7. Histopathology and immunohistochemistry (PCNA, TUNEL (terminal deoxynucleotidyl transferase dUTP nick end labeling), myeloperoxidase (MPO), Ki-67) were performed on baseline and day 7 wedge liver biopsies. Macroscopic liver regeneration was quantified by liver weight and volume measurements at sacrifice on postoperative day 7. The Mann-Whitney U test was used for continuous variables, with p < 0.05 considered statistically significant.

Results

Body weight, hepatectomy percentage, and intraoperative hemodynamics were comparable between the two groups. Mean arterial lactate at the end of ischemia (5.98 vs. 2.85 mmol/L, p = 0.005) and at 24 hours of reperfusion (2.10 vs. 1.11 mmol/L, p < 0.001) was significantly lower in the sildenafil group. ALT, AST, and LDH were significantly lower in the sildenafil group at 24 hours after reperfusion and at postoperative day 7 (all p ≤ 0.05). At six hours of reperfusion, TNF-α (4.84 vs. 11.17 pg/mL, p = 0.018) and IL-1 (3.09 vs. 7.47 pg/mL, p = 0.013) were lower in the sildenafil group, while IL-6 was higher (5.86 vs. 3.90 pg/mL, p = 0.017), consistent with a hepatoprotective IL-6 response. Histology also demonstrated decreased hepatocellular necrosis and a higher overall histopathological score in the control group (116 vs. 58, p = 0.025). Macroscopic regeneration of the remnant liver volume by postoperative day 7 was 22.5% in the sildenafil group versus 13.9% in controls (p = 0.002). One control animal in the control group died on postoperative day 3 from acute liver failure (mortality 14.3% vs. 0%).

Conclusions

In this porcine pilot study, perioperative oral sildenafil attenuated hepatocellular injury and the early systemic inflammatory response, and improved macroscopic liver regeneration after major hepatectomy with warm ischemia. These findings extend prior rodent observations into a clinically relevant large-animal model and support further translational evaluation of PDE-5 inhibition as a hepatoprotective strategy in major liver surgery and transplantation.

## Introduction

Hepatic ischemia-reperfusion (I/R) injury is a clinically important consequence of vascular inflow occlusion during liver resection and of cold ischemia during liver transplantation [1,2]. The pathophysiology involves intracellular ATP depletion and calcium overload during ischemia, followed by a burst of reactive oxygen species, mitochondrial dysfunction, neutrophil-driven sterile inflammation, and hepatocyte apoptosis and necrosis upon reperfusion [1,2]. After major hepatectomy, where the residual remnant must simultaneously withstand reperfusion injury and undertake regeneration, I/R injury is a recognized contributor to post-hepatectomy liver failure (PHLF) and to early postoperative morbidity and mortality [3,4].

Multiple strategies have been explored to mitigate hepatic I/R injury, including ischemic preconditioning (direct or remote), selective hypothermia, modulation of portal inflow, and a variety of pharmacological agents such as N-acetylcysteine, alprostadil, branched-chain amino acids, and corticosteroids [3-7]. Despite encouraging experimental data, none of these has translated into a robust standard of care, and the search for safer pharmacological adjuncts that protect the remnant liver while supporting regeneration continues [8].

Nitric oxide (NO) has emerged as a central regulator of the hepatic microcirculation during I/R. “Endogenous” NO produced by endothelial NO synthase counteracts endothelin-1-mediated sinusoidal vasoconstriction, preserves microvascular flow, and limits leukocyte-endothelial adhesion. However, excessive NO from inducible NO synthase during reperfusion can be cytotoxic [9-11]. NO acts largely through cyclic guanosine monophosphate (cGMP), which is rapidly degraded by phosphodiesterase-5 (PDE-5). Sildenafil is a potent and selective PDE-5 inhibitor that increases cGMP availability, promotes vasodilation, and has been shown to protect myocardium and hepatocytes from I/R injury in rodent models, partly through anti-apoptotic and anti-oxidant mechanisms [12,13]. In rats, perioperative sildenafil reduced alanine aminotransferase (ALT) and aspartate aminotransferase (AST) after hepatic I/R and accelerated regeneration after partial hepatectomy [14,15].

However, available data on sildenafil for hepatic I/R injury are limited and largely confined to small-rodent models that do not replicate the hemodynamic and regenerative challenge of major hepatectomy in clinical practice. Large-animal data are particularly important because porcine liver anatomy, vascular physiology, and operative scale are closer to the human ones, and a 65% hepatectomy in pigs has previously been used to study reperfusion injury after major liver resection [16,17]. The present study was designed to test whether perioperative oral sildenafil attenuates I/R injury, modulates the early systemic inflammatory response, and enhances macroscopic and microscopic liver regeneration in a porcine model of 65% hepatectomy combined with 45 minutes of normothermic ischemia.

## Materials and methods

Study design and animals

This was a prospective, controlled experimental study in 14 healthy male domestic pigs weighing 28-39 kg. Animals were randomized 1:1 to a control group (Group A, n = 7) and a sildenafil group (Group B, n = 7). Sample size was determined a priori on the basis of a previous experimental study in rats reporting an approximately 50% reduction in postoperative ALT and AST with sildenafil [14]; with α = 0.05 and β = 0.10, seven animals per group were required.

All animals had free access to food and water and were fasted for eight hours preoperatively. The protocol was approved by the institutional ethics committee of the National and Kapodistrian University of Athens School of Medicine under the auspices of the 4th Department of Surgery, Attikon University General Hospital, and experiments were conducted at the ELPEN Experimental and Research Center, Pikermi, Greece, in accordance with the Directive 2010/63/EU of the European Parliament and of the Council on the protection of animals used for scientific purposes and were reported in accordance with the ARRIVE 2.0 guidelines [18].

Anesthesia and perioperative care

Premedication consisted of intramuscular atropine 0.8 mg, ketamine 5 mg/kg, and midazolam 0.8 mg/kg. After ear-vein cannulation, anesthesia was induced with intravenous thiopental 3-5 mg/kg, pancuronium 0.1 mg/kg, and fentanyl 5 µg/kg, followed by endotracheal intubation and mechanical ventilation with a 60% O₂-air mixture and isoflurane 0.2-0.8%. Maintenance anesthesia and analgesia were provided by continuous infusion of fentanyl 2 mg, ketamine 500 mg, and rocuronium 200 mg in 1,000 mL of 5% dextrose at an initial rate of 50 mL/hour, titrated to the physiological needs of the animal subjects. Fluid replacement consisted of a mixture of 35% dextrose, lactated Ringer’s solution, and gelatin (Haemaccel®), targeting a central venous pressure of 6-8 mmHg and a systolic arterial pressure of 100-120 mmHg. The right external jugular vein and right internal carotid artery were cannulated for monitoring and sampling. Cefuroxime 750 mg was administered intravenously at induction and every six hours for the first 24 postoperative hours. No animal required intraoperative or postoperative transfusion, and no major bleeding events occurred during the study.

Surgical procedure and ischemia-reperfusion protocol

Through a midline laparotomy, a baseline wedge biopsy was obtained from the free edge of the liver. After full mobilization and dissection of the hepatoduodenal ligament, the Pringle maneuver was applied with an atraumatic vascular clamp. An extended left hepatectomy was then performed, removing the entire left and middle hepatic lobes to leave a remnant of approximately 35% of the original liver mass. Ligation of left portal pedicle structures and the left and middle hepatic veins preceded parenchymal transection, which was performed with the clamp-crushing technique; hemostasis of the cut surface was secured with continuous sutures. The Pringle clamp was maintained until 45 minutes of total hepatic ischemia had elapsed, after which it was released and the remnant inspected for bleeding or bile leak. The abdomen was closed with a continuous fascial suture that included the skin.

Animals were monitored for approximately two hours under mechanical ventilation, then extubated and returned to the holding facility for postoperative care that included scheduled opioid analgesia and daily wound checks. On postoperative day 7, a re-laparotomy was performed under analogous anesthesia. After macroscopic inspection of the liver remnant and a final wedge biopsy, animals were euthanized with a single bolus of intravenous potassium chloride, and the remaining liver was excised for weight and volume assessment.

Drug administration

Animals in Group B received oral sildenafil at a dose of 0.3 mg/kg every eight hours, beginning 24 hours before surgery and continuing until the day of sacrifice. The dose was selected based on the dose previously used in pharmacokinetic studies of sildenafil in pulmonary arterial hypertension, where it has been shown to be well tolerated without significant hemodynamic instability [19]. The oral route was preferred over intraperitoneal or intravenous administration in order to better approximate human pharmacokinetics.

Sampling and biochemical measurements

Arterial blood gases were obtained immediately after intubation (t1, baseline), at the start and end of the Pringle maneuver, and at 24 hours of reperfusion. Venous blood samples were obtained at baseline (immediately after laparotomy), 20 minutes after Pringle release, and at six, 12, and 24 hours of reperfusion. Samples were centrifuged at 4,000 rpm for 20 minutes and stored at -80°C until analysis. The following parameters were measured at predefined time points: ALT, AST and lactate dehydrogenase (LDH) (markers of hepatocellular injury); total bilirubin, prothrombin time (PT) and activated partial thromboplastin time (aPTT) (markers of synthetic and hepatocellular function); C-reactive protein, tumor necrosis factor-α (TNF-α), IL-1 and IL-6 (markers of systemic inflammation); and malondialdehyde (MDA) as a marker of lipid peroxidation. Cytokines were measured by the EURO/DPC chemiluminescence immunoassay; MDA was determined spectrophotometrically at 586 nm using the 1-methyl-2-phenylindole (NMPI) chromogenic assay [20].

Histopathology and immunohistochemistry

Wedge biopsies obtained at baseline and at sacrifice were fixed in 10% neutral buffered formalin for 12 hours, dehydrated, and paraffin-embedded. Sections of 4 µm were cut and stained with hematoxylin and eosin (H&E) for histopathological assessment of hepatocellular regeneration, degeneration, necrosis, sinusoidal dilatation, portal inflammation, and biliary/Kupffer cell hyperplasia. A semi-quantitative scoring system was applied for comparison purposes (Table 1).

**Table 1:** Semi-quantitative histopathological and immunohistochemical scoring system. Numerical scores are shown in parentheses. HPF: high-power field; MPO: myeloperoxidase

Variable	Mild	Moderate	Severe
Hepatocellular regeneration (binucleated cells per 1,000 hepatocytes)	≤10 (5)	10-20 (10)	>20 (15)
Hepatocellular necrosis (foci per lobule)	<2 foci (20)	3-5 foci (40)	>5 foci (60); bridging (80)
Portal inflammation	Mild (5)	Moderate (10)	Severe (15)
MPO-positive cells (per 3 HPF)	0-20 (5); 20-40 (10)	40-60 (15)	>60 (20)
Ki-67 staining	Mild (5)	Moderate (10)	Severe (15)

Immunohistochemistry was performed for proliferating cell nuclear antigen (PCNA) using a mouse monoclonal antibody with avidin-biotin-peroxidase detection and diaminobenzidine (DAB) chromogen. Apoptosis was assessed using the TUNEL (terminal deoxynucleotidyl transferase dUTP nick end labeling) method with digoxigenin-labelled dUTP, and myeloperoxidase (MPO) was detected using a 1% polyclonal rabbit antibody (Dako Pathology Products, Hamburg, Germany). Similarly, Ki-67-positive nuclei were identified using a monoclonal antibody with DAB detection. The percentage of PCNA-positive nuclei and TUNEL-positive nuclei was determined on counts of 1,000 nuclei per slide at ×40 magnification, in duplicate. MPO and Ki-67 were graded by positive cell counts per high-power field as described in Table 1.

Liver regeneration assessment

Macroscopic regeneration was determined at sacrifice on postoperative day 7. Preoperative liver mass was estimated using the formula proposed for porcine experimental models by Martínez de la Maza et al. [21]: liver mass (g) = 26.34232 × body mass (kg) - 1.270629 × length (cm) + 163.0076. The resected specimen was weighed immediately after hepatectomy, allowing calculation of the in situ remnant mass. Volume was derived using a published density-based formula assuming a hepatic density of 1.07 g/mL, a value corresponding to the median porcine liver density reported by Niehues et al. [22]. Liver regeneration was expressed as the percentage volume increase of the remnant between hepatectomy and postoperative day 7.

Statistical analysis

Continuous variables are presented as mean ± standard deviation (SD) and were compared between groups using the Mann-Whitney U test. Categorical variables were compared with the chi-square test. The log-rank test was used for subgroup time-to-event comparisons. A two-sided p-value of <0.05 was considered statistically significant. Analyses were performed with SPSS Statistics version 25.0 (IBM SPSS Statistics for Windows, IBM Corp., Armonk, NY).

## Results

Animal characteristics and intraoperative course

All 14 animals completed the operative protocol. Body weight (mean 33.1 kg in Group A vs. 31.7 kg in Group B), hepatectomy percentage (mean 65.22% vs. 65.02%), and intraoperative core temperature did not differ between groups. There were no major intraoperative bleeding events, and no animal received blood products. Mean arterial pressure (MAP) and heart rate during the early postoperative period showed minor but statistically significant differences favoring a slightly lower MAP (MAP 85.76 vs. 95.37 mmHg at 24 hours, p = 0.015) and a higher intraoperative heart rate (e.g., 126 vs. 110 bpm at the start of laparotomy, p = 0.003) in the sildenafil group, consistent with the known mild systemic vasodilator effect of the drug. These differences did not translate into hemodynamic instability.

Arterial lactate and metabolic parameters

Arterial lactate at the end of the Pringle maneuver was substantially lower in Group B (mean 2.85 vs. 5.98 mmol/L, p = 0.005), and remained lower at 24 hours of reperfusion (1.11 vs. 2.10 mmol/L, p < 0.001) (Table 2). Other arterial blood gas parameters (pH, pO₂, pCO₂, HCO₃⁻, electrolytes, hemoglobin) did not differ significantly between groups. Postoperative day 7 blood glucose was higher in the control group, but values remained within the upper normal range for porcine subjects, where in clinical cohorts, early postoperative hyperglycemia after liver resection has been associated with increased postoperative complications [23].

**Table 2:** Variables with statistically significant differences between the control and sildenafil groups. Values are means; p-values from the Mann-Whitney U test. The “U statistic” column reports the Mann-Whitney U test statistic for each comparison. ALT: alanine aminotransferase; AST: aspartate aminotransferase; bpm: beats per minute; IL: interleukin; LDH: lactate dehydrogenase; MAP: mean arterial pressure; TNF-α: tumor necrosis factor-α; U: Mann-Whitney U statistic

Variable	Control (Group A)	Sildenafil (Group B)	U statistic	p-value
Arterial blood gases				
Lactate at end of Pringle (mmol/L)	5.98	2.85	49	0.005
Lactate at 24 hours of reperfusion (mmol/L)	2.1	1.11	49	<0.001
Hemodynamics				
MAP at 24 hours of reperfusion (mmHg)	95.37	85.76	47	0.015
Heart rate, pre-laparotomy (bpm)	101	109	10	0.042
Heart rate, start of laparotomy (bpm)	110	126	5	0.003
Heart rate, start of Pringle (bpm)	133	141	8	0.026
Hepatocellular injury markers				
ALT at 24 hours of reperfusion (U/L)	111	79	48	0.003
ALT at postoperative day 7 (U/L)	70	50	40	0.002
AST at 6 hours of reperfusion (U/L)	229	153	46	0.016
AST at postoperative day 7 (U/L)	200	123	35	0.04
LDH at 24 hours of reperfusion (IU/L)	962	506	49	<0.001
LDH at postoperative day 7 (IU/L)	485	365	40	0.002
Inflammatory markers (six hours of reperfusion)				
TNF-α (pg/mL)	11.17	4.84	43	0.018
IL-1 (pg/mL)	7.47	3.09	47	0.013
IL-6 (pg/mL)	3.9	5.86	4	0.017
Histopathology				
Aggregate hepatocellular injury/regeneration score	116	58	37	0.025
Macroscopic regeneration				
Liver volume regeneration at day 7 (%)	13.9	22.5	4	0.002

Liver function and hepatocellular injury markers

ALT, AST, and LDH increased after reperfusion in both groups, peaking between six and 24 hours of reperfusion. Differences between groups reached statistical significance for ALT at 24 hours and at postoperative day 7 (mean 111 vs. 79 U/L, p = 0.003 and 70 vs. 50 U/L, p = 0.002), for AST at six hours and at day 7 (229 vs. 153 U/L, p = 0.016 and 200 vs. 123 U/L, p = 0.040), and for LDH at 24 hours and at day 7 (962 vs. 506 IU/L, p < 0.001 and 485 vs. 365 IU/L, p = 0.002), all favoring the sildenafil group (Table 2). Total bilirubin, PT, and aPTT were higher in the control group but did not reach statistical significance, with the exception of one control animal that developed clinically detectable jaundice and died on postoperative day 3 from acute liver failure.

Inflammatory markers

At six hours of reperfusion, TNF-α and IL-1 were significantly lower in the sildenafil group B (4.84 vs. 11.17 pg/mL, p = 0.018 and 3.09 vs. 7.47 pg/mL, p = 0.013, respectively). Conversely, IL-6 was significantly higher in Group B (5.86 vs. 3.90 pg/mL, p = 0.017), in line with its proposed hepatoprotective and pro-regenerative role. CRP and white cell count rose comparably in both groups. Plasma MDA concentrations did not differ significantly between groups, although elevated values persisted longer (>24 hours) in the control group.

Histopathology and immunohistochemistry

**Figure 1:**
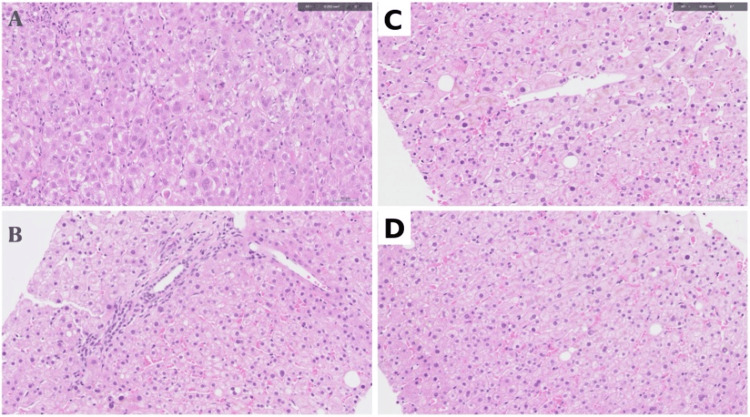
Representative hematoxylin and eosin (H&E) staining of postoperative day 7 wedge biopsies. (A) Marked hepatocellular degeneration in a control-group remnant. (B) Pronounced sinusoidal infiltration by aggregated leukocytes along a portal triad in a control-group remnant. (C) Generalized absence of leukocyte infiltration in a sildenafil-group remnant. (D) Mild degenerative changes in a sildenafil-group remnant. Original magnification ×20; scale bar = 50 µm.

Histopathological evaluation of postoperative day 7 biopsies showed greater hepatocellular regenerative activity, fewer necrotic foci, and milder portal inflammation in the sildenafil group (Figure 1). The aggregate histopathological injury/regeneration score was significantly lower in Group B (58 vs. 116, p = 0.025; Table 2). Immunohistochemical staining showed numerically higher PCNA expression and Ki-67 positivity in Group B and higher TUNEL-positive nuclear counts in Group A, but differences did not reach statistical significance, possibly because day-7 sampling captures a relatively late, equilibrated phase of regeneration, after the proliferative burst that occurs in the first postoperative days [24].

Macroscopic liver regeneration and animal survival

Mean macroscopic regeneration of remnant liver volume between hepatectomy and postoperative day 7 was significantly greater in the sildenafil group (22.5% vs. 13.9%, p = 0.002; Table 2). One animal in the control group (animal 1) died on postoperative day 3 from acute liver failure with jaundice and rising bilirubin, in the absence of macroscopic findings of steatosis, fibrosis, or cirrhosis on autopsy and despite a hepatectomy ratio (66.4%) within the planned safety margin (mortality 14.3% in Group A vs. 0% in Group B). All other animals were alive and clinically well at the time of scheduled re-laparotomy.

Time-course of injury and inflammatory markers

**Figure 2:**
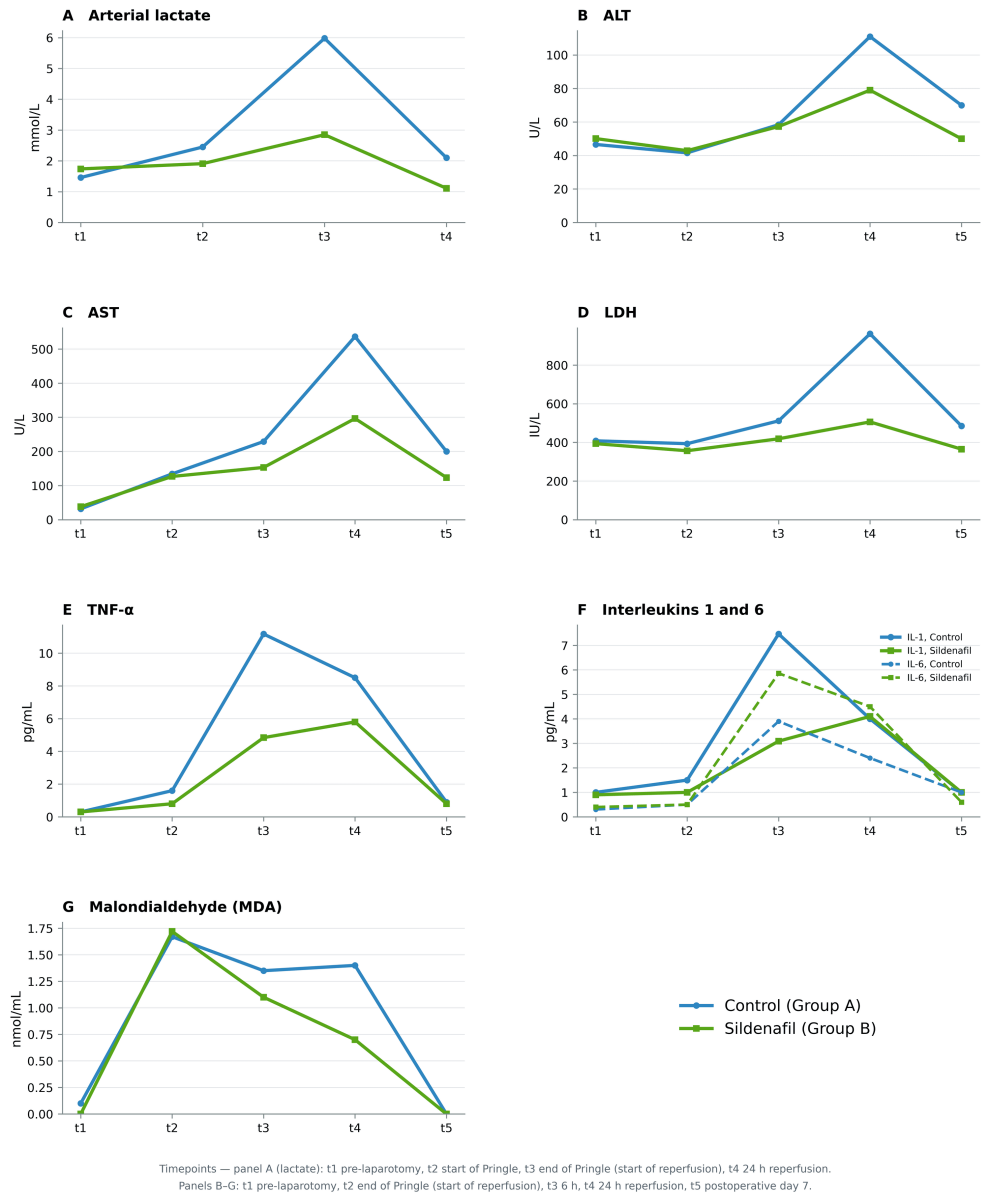
Time-course of the principal biochemical and inflammatory markers (mean values) in the control (Group A) and sildenafil (Group B) groups. (A) Arterial lactate; (B) ALT; (C) AST; (D) LDH; (E) TNF-α; (F) interleukins 1 and 6 (IL-1, solid lines; IL-6, dashed lines); (G) malondialdehyde (MDA). For panel A (arterial blood gas), timepoints are as follows: t1, pre-laparotomy; t2, start of the Pringle maneuver; t3, end of the Pringle maneuver (start of reperfusion); t4, 24 hours of reperfusion. For panels B-G, timepoints are as follows: t1, pre-laparotomy; t2, end of the Pringle maneuver (start of reperfusion); t3, six hours of reperfusion; t4, 24 hours of reperfusion; t5, postoperative day 7. ALT: alanine aminotransferase; AST: aspartate aminotransferase; IL: interleukin; LDH: lactate dehydrogenase; TNF-α: tumor necrosis factor-α

The temporal profiles of the principal biochemical and inflammatory markers are summarized in Figure 2. Arterial lactate (Figure 2A) rose in both groups during ischemia but peaked markedly higher in the control group at the end of the Pringle maneuver, before declining by 24 hours of reperfusion. The hepatocellular injury markers ALT, AST, and LDH (Figures 2B-2D) followed a characteristic trajectory: a transient dip at the end of ischemia, a steep rise to a peak at 24 hours of reperfusion, and partial recovery by postoperative day 7. Throughout the reperfusion and recovery phases, the control group maintained higher values, and the separation between groups for AST and LDH was already evident from six hours of reperfusion onward and persisted to day 7.

The inflammatory markers showed a divergent pattern (Figures 2E-2G). TNF-α (Figure 2E) peaked at six hours of reperfusion in both groups but reached approximately twice the concentration in the control group. The interleukin profiles (Figure 2F) were informative: IL-1 peaked at six hours and was higher in the control group, whereas IL-6 - a cytokine with a recognized hepatoprotective and pro-regenerative role - was higher in the sildenafil group around the same window. MDA, a marker of lipid peroxidation (Figure 2G), rose comparably in both groups after reperfusion but remained elevated for longer in the control group, consistent with more sustained oxidative stress.

## Discussion

In this porcine pilot study, perioperative oral sildenafil administered at 0.3 mg/kg every eight hours, beginning 24 hours before a 65% hepatectomy with 45 minutes of warm ischemia, was associated with attenuated hepatocellular injury, dampened early systemic inflammatory response, and a clinically meaningful improvement in macroscopic liver regeneration on postoperative day 7. To our knowledge, this is one of the few large-animal studies to test sildenafil in a model that simultaneously captures both major liver resection and warm ischemia, and the resulting hemodynamic, biochemical, and regenerative challenge is closer to the clinical context of major hepatectomy and donation-after-circulatory-death (DCD) liver transplantation than previously published rodent work [14,15].

The most consistent biochemical signal was a sustained reduction in ALT, AST, and LDH from six hours of reperfusion through postoperative day 7. Clinical studies have shown that early postoperative AST and, to a lesser extent, ALT, are independent predictors of PHLF and 90-day mortality [25]. Postoperative LDH has likewise been associated with worse outcomes after major hepatectomy [26]. The persistent suppression of these markers in the sildenafil group, beyond the immediate reperfusion phase and through the regenerative window, suggests that PDE-5 inhibition not only blunts the initial reperfusion insult but may also support the recovery phase. This is biologically plausible: increased intracellular cGMP through PDE-5 inhibition activates protein kinase G, opens mitochondrial K⁺-ATP channels, relaxes vascular smooth muscle, and may also exert direct anti-oxidant and anti-apoptotic effects independent of the NO-cGMP axis [12,27].

Arterial lactate results also provided a complementary perspective. Mean lactate of 5.98 mmol/L at the end of the Pringle maneuver in the control group is in the range associated, in clinical cohorts, with a substantially elevated risk of PHLF and complications [28-30]. The marked reduction with sildenafil to 2.85 mmol/L, with values remaining lower at 24 hours of reperfusion, is consistent with improved hepatic microcirculatory flow and oxygen delivery, the proposed primary mechanism of PDE-5 inhibition in the I/R setting [12]. The systemic vasodilatory and hypotensive effect of sildenafil could, in principle, reduce hepatic perfusion pressure and thereby worsen, rather than protect, the remnant liver. Our data argue against this in the present model: although MAP was modestly lower in the sildenafil group at 24 hours (85.76 vs. 95.37 mmHg, p = 0.015), this was accompanied by lower arterial lactate and lower hepatocellular injury markers. This dissociation between a small fall in systemic pressure and improved markers of hepatic oxygenation is mechanistically coherent: by raising cGMP, PDE-5 inhibition preferentially relaxes the hepatic sinusoidal microcirculation and counteracts endothelin-1-mediated vasoconstriction, so that effective microvascular perfusion is preserved or improved despite the modest systemic effect. Overall, the systemic vasodilatory effect of the drug at this dose was well tolerated and did not undermine its hepatoprotective signal.

The cytokine profile at six hours of reperfusion was also informative regarding the sildenafil effect. The early peak of TNF-α and IL-1, recognized drivers of the post-reperfusion sterile inflammatory cascade [1,2], was significantly attenuated by sildenafil. In contrast, IL-6 was significantly higher in the sildenafil group at the same time point. Although IL-6 is a pro-inflammatory cytokine, in the context of liver regeneration after hepatectomy, it is a key mitogenic signal acting via STAT3 and is associated with hepatoprotection rather than injury. The dissociation observed here - lower TNF-α and IL-1 with higher IL-6 - is consistent with a redirection of the early postoperative inflammatory response toward a more regenerative phenotype rather than a generalized immunosuppression.

This biochemical and inflammatory signal was matched by the histological and macroscopic findings. The aggregate histopathological score was significantly lower in the sildenafil group (58 vs. 116, p = 0.025), with less hepatocellular necrosis and milder portal inflammation. Macroscopic remnant liver regeneration at postoperative day 7 was 22.5% in the sildenafil group versus 13.9% in controls (p = 0.002). This convergent pattern of less injury and inflammation, and more regeneration, supports a true biological effect rather than an isolated laboratory finding. The single death from acute liver failure in the control group, although insufficient to demonstrate a survival difference in such a small cohort, is in line with the rest of the data and underscores the clinical relevance of the observed differences.

Differences in PCNA, TUNEL, MPO, and Ki-67 trended in the same direction but did not reach statistical significance. Two explanations are plausible. First, the timing of the final biopsy (postoperative day 7) likely captures a late, equilibrated phase of regeneration in which proliferation indices may have already plateaued; earlier sampling (e.g., postoperative days 2-3) would probably be more informative for these markers. Second, with seven animals per group, the study is powered for the largest effects (liver function tests (LFTs), regenerative volume) but underpowered for the more variable immunohistochemical endpoints. These considerations should inform the design of confirmatory studies.

Several limitations should be acknowledged in this experimental study. The sample size of 14 animals is considered relatively small, although it was sized on the basis of expected differences in transaminases [14]. The model uses healthy young pigs without underlying chronic liver disease, steatosis, or cirrhosis, which differs from many clinical patients undergoing major hepatectomy for hepatocellular carcinoma or after neoadjuvant chemotherapy, mainly in colorectal liver metastases. This distinction is particularly relevant for sildenafil, since in clinical practice the drug can cause systemic hypotension and is used with caution in patients with established liver disease, in whom altered hepatic clearance and portal hemodynamics may modify both its pharmacokinetics and its hemodynamic effects. Our findings in healthy animals, therefore, cannot be assumed to translate directly to such patients, and dedicated safety and dose-finding evaluation will be required before clinical use. Sildenafil was administered orally to mimic clinical pharmacokinetics, but bioavailability was not directly measured. Additionally, intraoperative blood loss, a recognized determinant of both I/R injury and regeneration, was not formally quantified as a predefined endpoint. We can confirm that no animal required transfusion and that no major bleeding events occurred, and we recommend that measured blood loss be recorded as a standardized endpoint in future studies. Finally, follow-up was limited to seven days, sufficient to capture early regeneration but not longer-term remodeling. Despite these limitations, the consistency of the findings across hemodynamic, biochemical, inflammatory, histological, and macroscopic readouts supports a genuine hepatoprotective and pro-regenerative effect of perioperative sildenafil in this model.

Clinically, several settings could plausibly benefit from PDE-5 inhibition: extended hepatectomy with prolonged inflow occlusion, donor and recipient management in DCD liver transplantation, where I/R injury and microcirculatory dysfunction are central to graft outcomes [30], and patients with marginal future liver remnants, where regenerative reserve is limited. Sildenafil has a well-characterized human safety profile and is widely used for pulmonary hypertension; this lowers the translational barrier compared with novel agents. The next steps should include confirmatory large-animal studies with earlier histological sampling and longer follow-up, dose-response work, mechanistic studies of the NO-cGMP axis and mitochondrial K⁺-ATP channels in the porcine liver, and ultimately a carefully designed clinical pilot in selected hepatectomy patients.

## Conclusions

In a porcine model of 65% hepatectomy with 45 minutes of warm ischemia, perioperative oral sildenafil 0.3 mg/kg every eight hours, started 24 hours before surgery and continued for seven days postoperatively, attenuated hepatocellular injury (lower ALT, AST and LDH from early reperfusion through postoperative day 7), dampened the early TNF-α and IL-1 response while preserving IL-6, reduced histological evidence of hepatocellular necrosis and inflammation, and significantly improved macroscopic remnant liver regeneration. These findings extend prior rodent observations into a clinically relevant large-animal model and support further translational work to evaluate PDE-5 inhibition as a hepatoprotective adjunct in major hepatectomy and liver transplantation.
